# Neutrophil extracellular traps induced by the hypoxic microenvironment in gastric cancer augment tumour growth

**DOI:** 10.1186/s12964-023-01112-5

**Published:** 2023-05-01

**Authors:** Jiacheng Li, Yu Xia, Biying Sun, Nanbei Zheng, Yang Li, Xuehan Pang, Fan Yang, Xingwang Zhao, Zhiwu Ji, Haitao Yu, Fujun Chen, Xuemei Zhang, Bin Zhao, Jiaqi Jin, Shifeng Yang, Zhuoxin Cheng

**Affiliations:** 1 Department of General Surgery, The First Affiliated Hospital of Jiamusi University, Heilongjiang Province, Jiamusi, 154000 China; 2 Digestive Disease Center, The First Affiliated Hospital of Jiamusi University, Heilongjiang Province, Jiamusi, 154000 China; 3 Department of Gastroenterology, The First Affiliated Hospital of Jiamusi University, Heilongjiang Province, Jiamusi, 154000 China; 4 Department of Neurosurgery, The Second Affiliated Hospital of Harbin Medical University, Heilongjiang Province, Harbin, 150001 China; 5 Department of General Surgery, The Second Affiliated Hospital of Harbin Medical University, Heilongjiang Province, Harbin, 150001 China

**Keywords:** Neutrophil extracellular traps, Gastric cancer, Hypoxia, Tumour immune microenvironment, HMGB1

## Abstract

**Background:**

Inflammation-related predisposition to cancer plays an essential role in cancer progression and is associated with poor prognosis. A hypoxic microenvironment and neutrophil infiltration are commonly present in solid tumours, including gastric cancer (GC). Neutrophil extracellular traps (NETs) have also been demonstrated in the tumour immune microenvironment (TIME), but how NETs affect GC progression remains unknown. Here, we investigated the role of NET formation in the TIME and further explored the underlying mechanism of NETs in GC tumour growth.

**Methods:**

Hypoxia-induced factor-1α (HIF-1α), citrulline histone 3 (citH3) and CD66b expression in tumour and adjacent nontumor tissue samples was evaluated by western blotting, immunofluorescence and immunohistochemical staining. The expression of neutrophil-attracting chemokines in GC cells and their hypoxic-CM was measured by qRT‒PCR and ELISA. Neutrophil migration under hypoxic conditions was evaluated by a Transwell assay. Pathway activation in neutrophils in a hypoxic microenvironment were analysed by western blotting. NET formation was measured in vitro by immunofluorescence staining. The protumour effect of NETs on GC cells was identified by Transwell, wound healing and cell proliferation assays. In vivo, an lipopolysaccharide (LPS)-induced NET model and subcutaneous tumour model were established in BALB/c nude mice to explore the mechanism of NETs in tumour growth.

**Results:**

GC generates a hypoxic microenvironment that recruits neutrophils and induces NET formation. High mobility group box 1 (HMGB1) was translocated to the cytoplasm from the nucleus of GC cells in the hypoxic microenvironment and mediated the formation of NETs via the toll-like receptor 4 (TLR4)/p38 MAPK signalling pathway in neutrophils. HMGB1/TLR4/p38 MAPK pathway inhibition abrogated hypoxia-induced neutrophil activation and NET formation. NETs directly induced GC cell invasion and migration but not proliferation and accelerated the augmentation of GC growth by increasing angiogenesis. This rapid tumour growth was abolished by treatment with the NET inhibitor deoxyribonuclease I (DNase I) or a p38 MAPK signalling pathway inhibitor.

**Conclusions:**

Hypoxia triggers an inflammatory response and NET formation in the GC TIME to augment tumour growth. Targeting NETs with DNase I or HMGB1/TLR4/p38 MAPK pathway inhibitors is a potential therapeutic strategy to inhibit GC progression.

Video Abstract

**Supplementary Information:**

The online version contains supplementary material available at 10.1186/s12964-023-01112-5.

## Background

Gastric cancer (GC) is one of the most common gastrointestinal tumours in the world and is the third leading cause of death in cancer patients [1]. More than one million new cases of GC are diagnosed every year worldwide, and most of them are in an advanced stage at diagnosis, resulting in increasingly prominent social health problems [2]. The prognosis of advanced GC is poor, with an estimated relative five-year survival rate of less than 30% [3, 4]. At present, radical D2 resection is still the first choice to cure GC. However, once the tumour progresses, for example, after the development of local recurrence, distant metastasis or cancer-associated thrombosis (CAT), no surgical treatment, chemotherapy, targeted therapy or immunotherapy can effectively prolong the survival time of patients [5, 6].

Inflammation is one of the established hallmarks of tumours. Neutrophils are the cells on the front line of pathogen defence and have been implicated as one of the principal cell types in the tumour immune microenvironment (TIME), participating in almost every step of tumour development and progression [7, 8]. Recently, accumulating evidence has started to support the idea that tumour-associated neutrophils (TANs) play a role in tumour progression via the formation of neutrophil extracellular traps (NETs), which are composed of extracellular chromatin decorated with citrulline histone H3 (citH3) together with antimicrobial protein granules and enzymes, such as neutrophil elastase (NE) and myeloperoxidase (MPO), in the TIME [9, 10]. Since the initial study of NETs in inflammatory diseases as a mechanism of microbe trapping and killing [11], NETs have also been implicated in cancer cell proliferation, metastasis and CAT [12–14]. Our previous study reported that NETs participate in the development of CAT in patients with GC and further showed that NETs can induce GC cell migration and invasion through epithelial-mesenchymal transition (EMT) [15–18]. In addition, other studies showed that NETs can directly promote cancer cell proliferation in diffuse large B-cell lymphoma (DLBCL) and colorectal cancer (CRC) [19, 20]. Although the prometastatic role of NETs has been found in many types of cancer, their effect on cancer cell proliferation is unknown.

Solid tumours, including GC, typically develop in hostile microenvironments, but cancer cells continue to exhibit enhanced growth [21, 22]. Hypoxia is the most common environmental condition in the tumour microenvironment, accompanied by rapid growth and predisposition to inflammatory responses. The current evidence described above indicates that highly proliferative tumours quickly outgrow their blood supply, leading to hypoxia [23, 24]. Subsequently, hypoxia-induced cancer cell death releases damage-associated molecular patterns (DAMPs), such as high mobility group box 1 (HMGB1), which recruit immune cells into the TIME [10, 25]. Recently, it has been reported that NETs significantly accumulate in liver tissue when animals undergo liver ischaemia/reperfusion (I/R), which is considered to induce hypoxia, and contribute to cancer cell metastasis [26]. Given the well-described but poorly understood association between hypoxia and NET formation, we sought to determine whether the hypoxic microenvironment plays a role in NET formation in GC patients. In addition, the mechanism of NETs in tumour growth in GC requires more detailed study.

Our central hypothesis is that hypoxia in GC tumours induces neutrophil recruitment and NET formation in the TIME; in turn, NETs accelerate the augmentation of GC growth. Thus, we deeply investigated the molecular mechanism of NET formation in the TIME of GC. Moreover, we showed the role of NETs in tumour growth both in vitro and in vivo. In general, our study may identify a new approach for the treatment of GC.

## Results

### More NETs accumulate in GC, and NET formation is correlated with cancer-specific outcomes

**Fig. 1:**
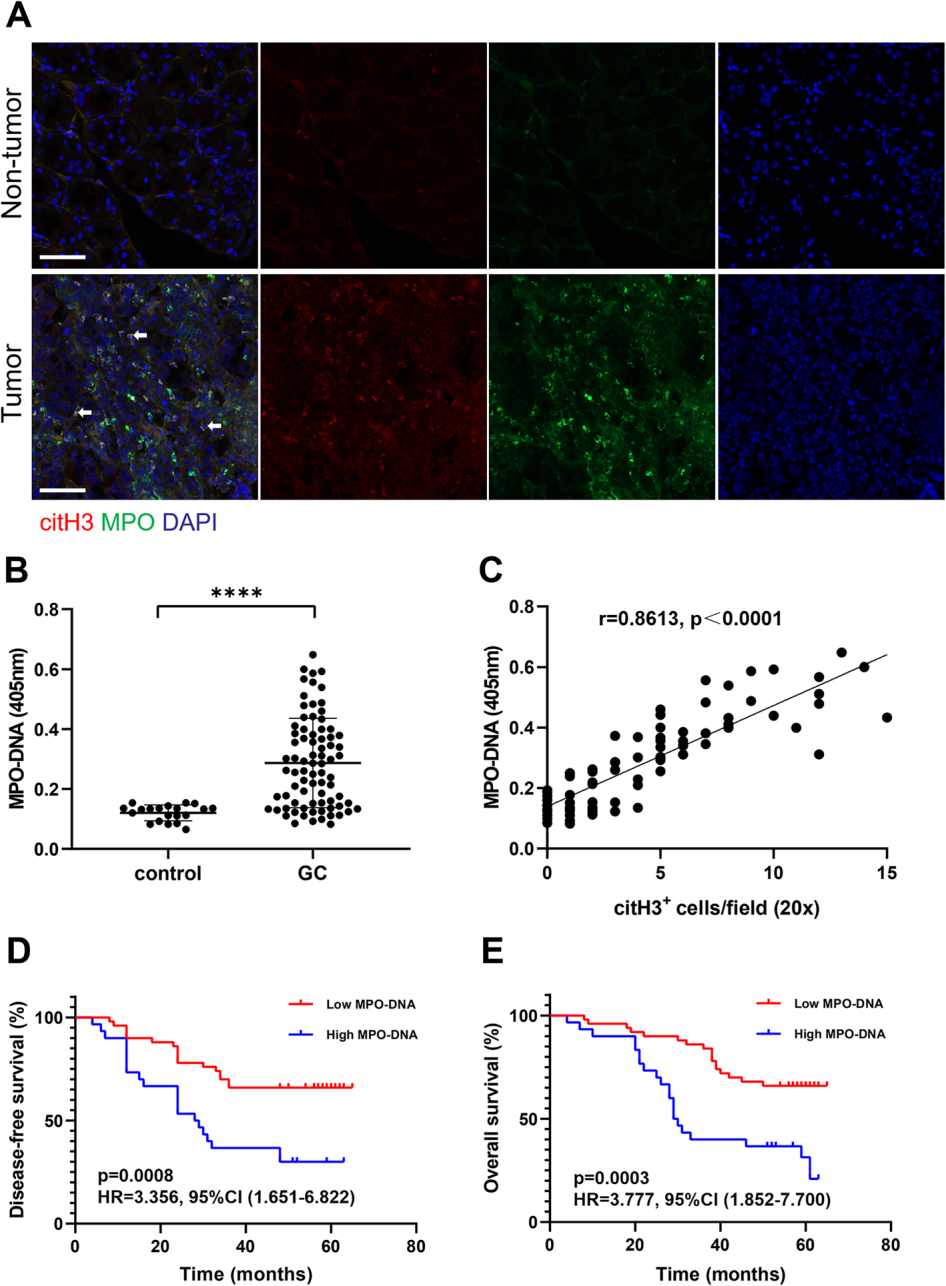
NET levels were higher in tumour tissue samples and correlated with poor prognosis in GC patients. **A**, Representative images of NETs in tumour tissue samples and nontumor tissue samples from the same GC patients detected with MPO and citH3 staining. The white arrows indicate bona fide NETs. Magnification: 20 × . Scale bars: 50 μm. **B**, The levels of MPO-DNA in the circulation of GC patients and healthy individuals were measured by ELISA. **C,** NETs in tumour tissue samples were positively correlated with plasma MPO-DNA levels. **D** and** E**, GC patients with higher plasma MPO-DNA levels (*n* = 30) had poorer DFS and OS than those with lower MPO-DNA levels (*n* = 50). Log-rank test and univariate Cox regression analysis, as indicated. All values are the means ± SDs. ****P < 0.0001

Previous studies reported that NET formation was associated with poor prognosis in cancer patients, including those with breast cancer, liver cancer and pancreatic cancer [12, 26, 27]. In this study, histopathologic analysis of tissues collected from 80 patients with GC undergoing radical D2 resection showed significantly increased NET formation (MPO^+^/citH3^+^ cells) in tumour tissue samples compared with nontumor tissue samples from the same patient (Fig. 1A). Furthermore, the preoperative serum levels of MPO-DNA were significantly increased in patients with GC compared with healthy individuals (Fig. 1B). Interestingly, the levels of plasma MPO-DNA positively correlated with the number of citH3-positive cells in tumour tissues (*r* = 0.8613, *p* < 0.0001, Fig. 1C). In addition, we analysed the DFS and OS of GC patients with preoperative plasma MPO-DNA levels above or below the median. We found that a high plasma level of MPO-DNA was associated with markedly worse DFS (HR: 3.356, 95% CI: 1.651–6.822, *p* = 0.0008, Fig. 1D) and OS (HR: 3.777, 95% CI: 1.852–7.700, *p* = 0.0003, Fig. 1E) using univariate analysis. Of note, multivariate Cox regression analysis revealed that the plasma level of MPO-DNA was an independent predictor of both DFS and OS after adjustment for other known prognostic variables, including TNM stage, size of the largest lesion and CEA level (DFS, HR: 1.328, 95% CI: 1.112–4.964, *p* = 0.003; OS, HR: 2.299, 95% CI: 1.102–5.875, *p* = 0.007). Taken together, our data showed that NETs accumulate in the tissue and blood of patients with GC and correlate with poor DFS and OS.

### GC generates a hypoxic microenvironment that recruits neutrophils and induces NET formation

**Fig. 2:**
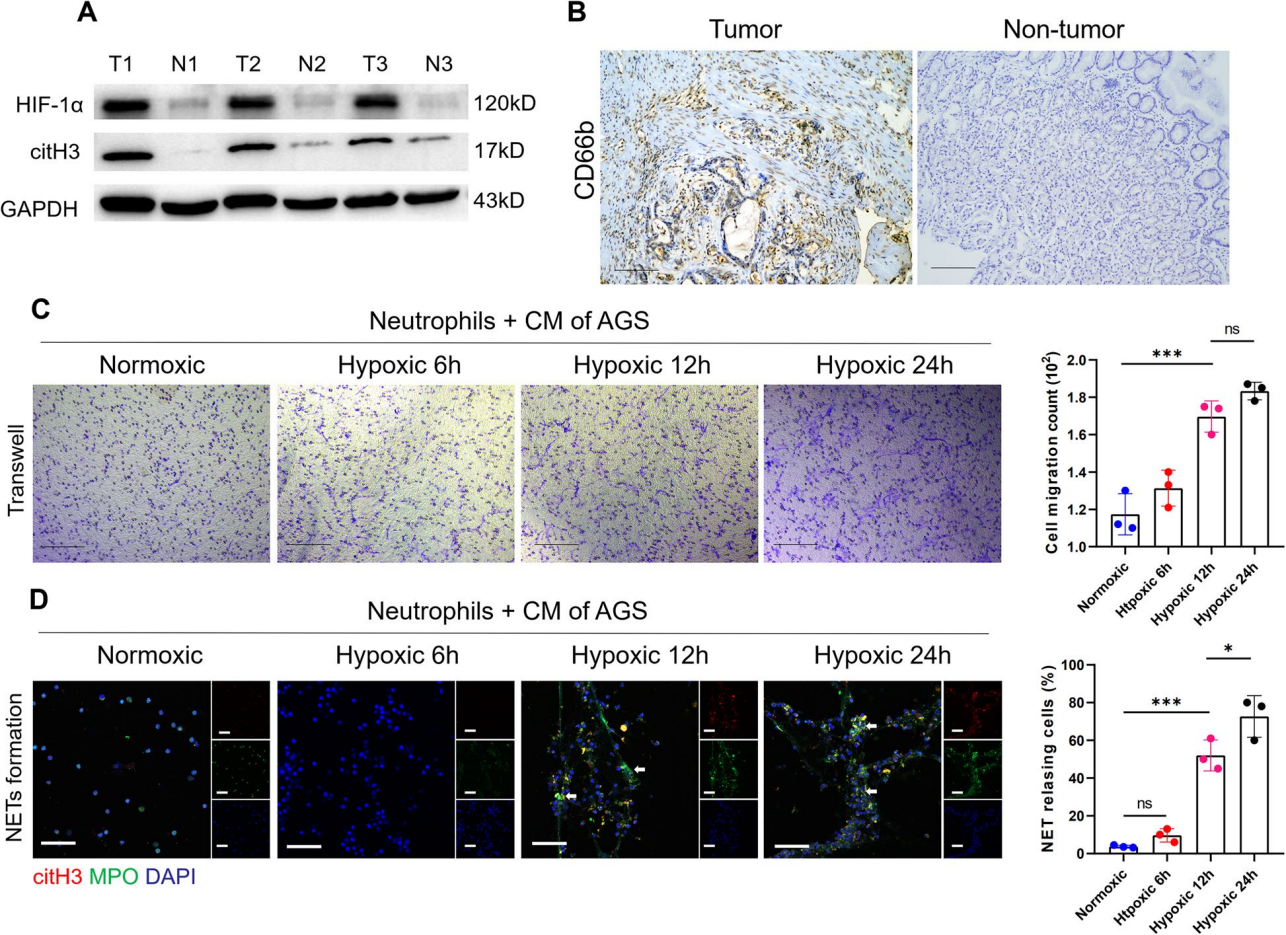
The hypoxic microenvironment of GC resulted in neutrophil recruitment and induction of NET formation. **A**, The expression of HIF-1α and citH3 was markedly increased in tumour tissues compared with nontumor tissues from GC patients.** B**, The expression of CD66b in tumour tissue and nontumor tissue samples from the same GC patient was evaluated by IHC staining. Magnification: 10 × . Scale bars: 100 μm. **C**, Neutrophils isolated from healthy individuals were cocultured with hypoxic-CM from GC cells, and neutrophil migration was measured by a Transwell assay. **D**, Representative images of NET formation by neutrophils incubated with hypoxic-CM from GC cells detected with MPO and citH3 staining. The white arrows indicate bona fide NETs. The percentage of NET releasing cells was defined as the ratio of the calculated NET releasing neutrophils to the total number of neutrophils. Magnification: 20 × . Scale bars: 50 μm. All values are the means ± SDs. ns = not significant, *p < 0.05 and ***P < 0.001

Since the preoperative plasma levels of NETs are correlated with poor prognosis in GC patients, as shown above, we investigated the mechanism of NET formation in GC. Hypoxia is commonly present in rapidly growing tumours and has been linked to tumour progression. A previous study reported that NETs markedly accumulated in liver tissue under ischaemia/reperfusion (I/R) conditions, which are considered to induce hypoxia, subsequently promoting tumour metastasis [28]. To investigate the relationship between hypoxia and NET formation in GC, we examined the expression of HIF-1α and citH3 in human GC tissue samples and the corresponding nontumor tissue samples by western blotting. We showed that the expression of HIF-1α and citH3 was significantly increased in GC tissues compared with nontumor tissues from the same patient (Fig. 2A). Furthermore, neutrophil infiltration was demonstrated in tissue samples of patients with GC by IHC staining for CD66b (Fig. 2B). Since neutrophil accumulation and NET formation were present in the hypoxic GC microenvironment, we examined whether hypoxia plays a role in neutrophil recruitment and NET formation. AGS cells were cultured under normoxic or hypoxic conditions, and their CM was incubated with neutrophils isolated from healthy individuals. Then, neutrophil recruitment in the GC microenvironment was assessed using a migration assay with Transwell chambers, and NET formation was evaluated by IF staining. We found that neutrophils migrated more quickly when cultured with hypoxic-CM than when cultured with normoxic-CM (Fig. 2C). Furthermore, the results showed that hypoxic-CM from GC cells potently stimulated NET formation (Fig. 2D). Although neutrophils cocultured with normoxic-CM from GC cells exhibited some NET release, it was significantly reduced compared with that of neutrophils cocultured with hypoxic-CM.

### Hypoxic-CM activates the p38 MAPK pathway in neutrophils to release NETs

**Fig. 3:**
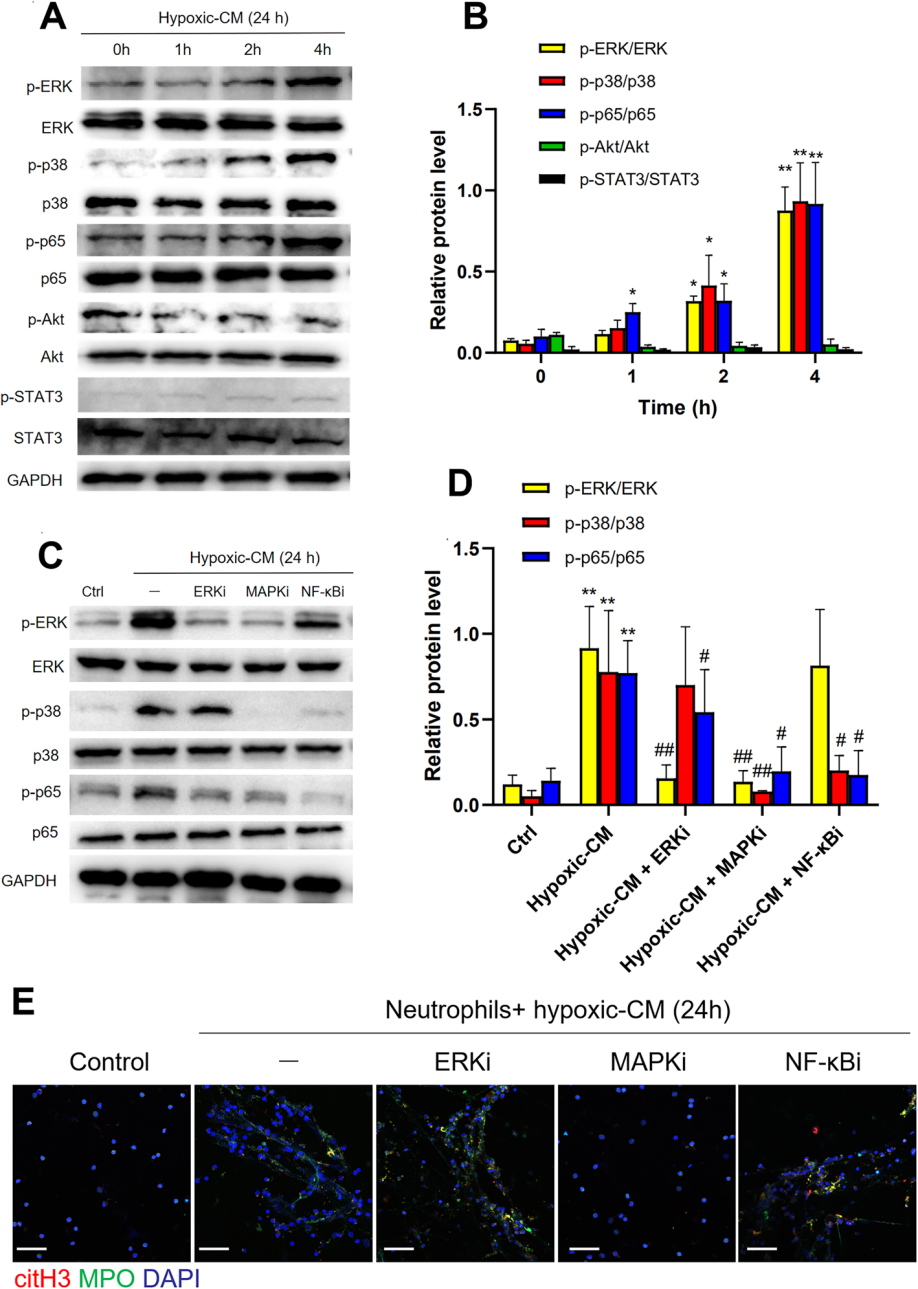
Hypoxic CM from GC cells induced NET formation through activation of the p38 MAPK pathway in neutrophils. **A** and** B**, Western blot analysis of p-ERK, p-p38, p-p65, p-Akt, and p-STAT3 levels in neutrophils cocultured with hypoxic-CM from GC cells for 2 h. **C-E**, Neutrophils were pretreated with inhibitors of the ERK, p38 MAPK, and p65 NF-кB pathways prior to incubation with hypoxic-CM from GC cells. The protein levels of p-ERK, p-p38, and p-p65 in neutrophils were measured by western blotting, and NET formation by neutrophils was evaluated with MPO and citH3 staining. Magnification: 20 × . Scale bars: 50 μm. All values are the means ± SDs. ns = not significant, *p < 0.05 and **P < 0.01 compared to the control group; #*p* < 0.05 and ##*p* < 0.01 compared to the hypoxic-CM (24 h) group

To clarify the mechanism by which NETs release neutrophils under hypoxic conditions, neutrophils isolated from healthy individuals were cocultured with hypoxic-CM from AGS cells, and the activation states of various pathways were determined by western blotting. The results showed that the protein levels of p-p65 NF-кB, p-p38 MAPK and p-ERK were significantly increased in the hypoxic-CM group compared with the control group (Fig. 3A and B). In addition, the levels of p-STAT3 and p-Akt were barely increased in neutrophils that were treated with hypoxic-CM (Fig. 3A and B). Furthermore, we pretreated neutrophils in the presence of a p65 NF-кB, p38 MAPK or ERK pathway inhibitor before coculture with hypoxic-CM from AGS cells. Notably, the p38 MAPK inhibitor reversed the hypoxic-CM-induced activation of the p65 NF-кB and ERK pathways (Fig. 3C and D). We also found that only the p38 MAPK inhibitor inhibited the activation of neutrophils and that NET formation was significantly decreased after treatment with this inhibitor compared with the other signalling pathway inhibitors (Fig. 3E), indicating that the p38 MAPK pathway plays a role in NET formation in a hypoxic microenvironment.

### HMGB1 is translocated to the cytoplasm and participates in NET formation in a hypoxic environment

**Fig. 4:**
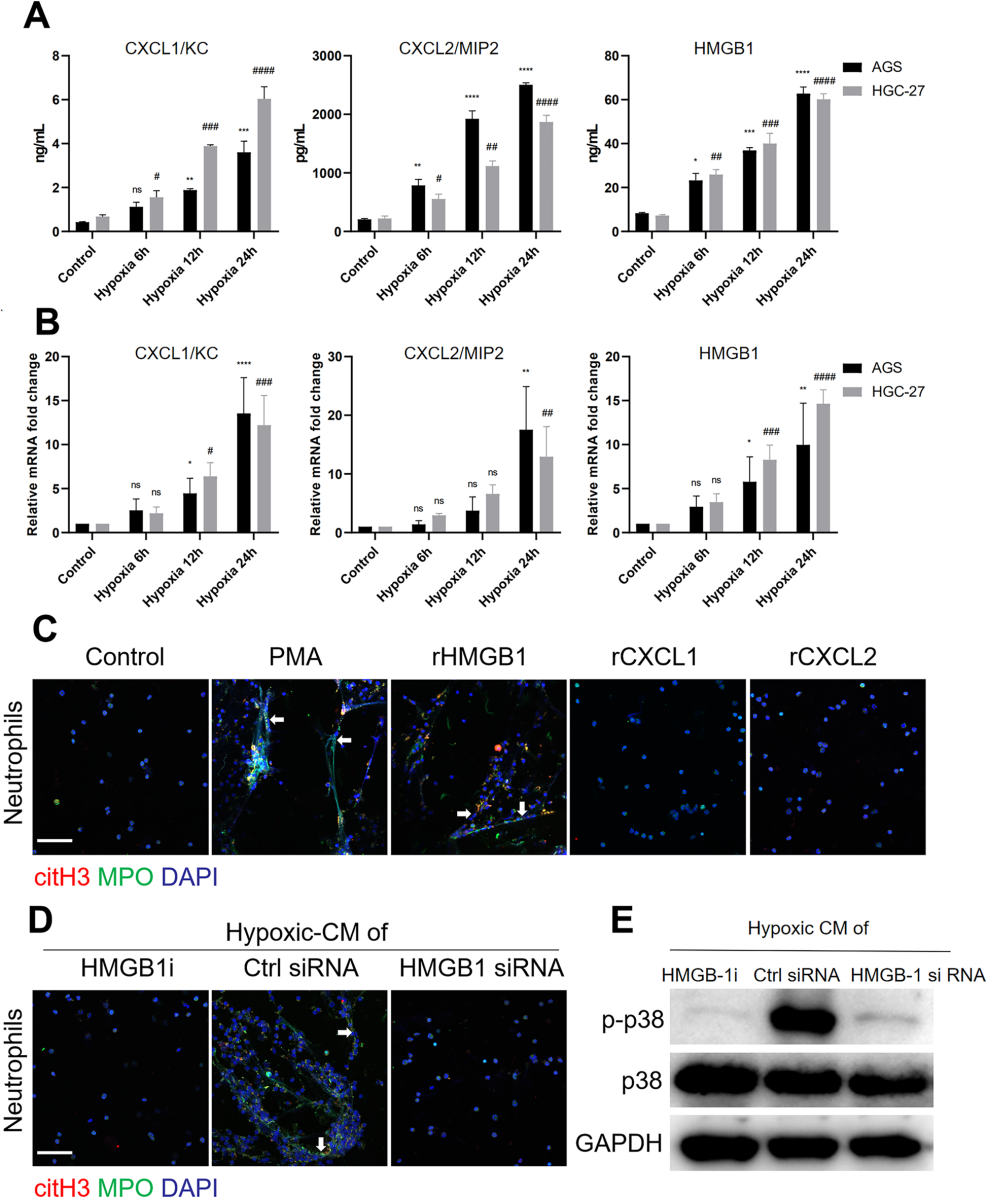
Neutrophil-attracting chemokine levels were significantly increased in GC cells incubated under hypoxic conditions. **A** and** B**, GC cells were incubated under hypoxic conditions, and the levels of neutrophil-attracting chemokines were measured by ELISA and qRT‒PCR. **C**, Neutrophils isolated from healthy individuals were cocultured with recombinant HMGB1, recombinant CXCL1, recombinant CXCL2 or PMA for 3 h, and NET formation was detected with MPO and citH3 staining. Images were acquired using confocal microscopy. **D** and** E**, Neutrophils were cocultured with hypoxic-CM from GC cells treated with an HMGB1 inhibitor or with hypoxic-CM from HMGB1-silenced GC cells. NET formation was evaluated by IF staining, and the level of p-p38 in neutrophils was measured by western blotting. Magnification: 20 × . Scale bars: 50 μm. All values are the means ± SDs. ns = not significant, *P < 0.05, **P < 0.01, ***P < 0.001, ****P < 0.0001 compared to control AGS cells; #*P* < 0.05, ##*P* < 0.01, ###*P* < 0.001, ####*P* < 0.0001 compared to control HGC-27 cells

To determine the mechanism by which a hypoxic microenvironment induces neutrophil migration and NET formation, we next examined the cellular chemokines in the hypoxic-CM of GC cells. We found that the levels of the neutrophil-attracting chemokines CXCL1/KC, CXCL2/MIP2 and HMGB1 in the hypoxic-CM of GC cells were significantly increased compared with those in the normoxic-CM, as shown by ELISA (Fig. 4A). In addition, the mRNA expression of CXCL1/KC, CXCL2/MIP2 and HMGB1 was increased when GC cells were cultured under hypoxic conditions (Fig. 4B). Indeed, NET release was observed when neutrophils were cocultured with recombinant HMGB1 or with PMA as a positive control but not with recombinant CXCL1 and CXCL2 (Fig. 4C). The addition of an HMGB1 antagonist to hypoxic-CM from GC cells markedly inhibited NET formation. Furthermore, we silenced HMGB1 expression in AGS cells by a gene-specific siRNA and then tested the ability of hypoxic-CM to induce NET formation. Compared to the hypoxic-CM from GC cells transfected with control siRNA, hypoxic-CM from HMGB1-silenced AGS cells resulted in significantly decreased NET formation by neutrophils derived from healthy controls. Similarly, hypoxic-CM from HMGB1 knockdown AGS cells had a decreased ability to activate the p38 MAPK pathway in neutrophils compared with hypoxic-CM from control siRNA-transfected AGS cells.

**Fig. 5:**
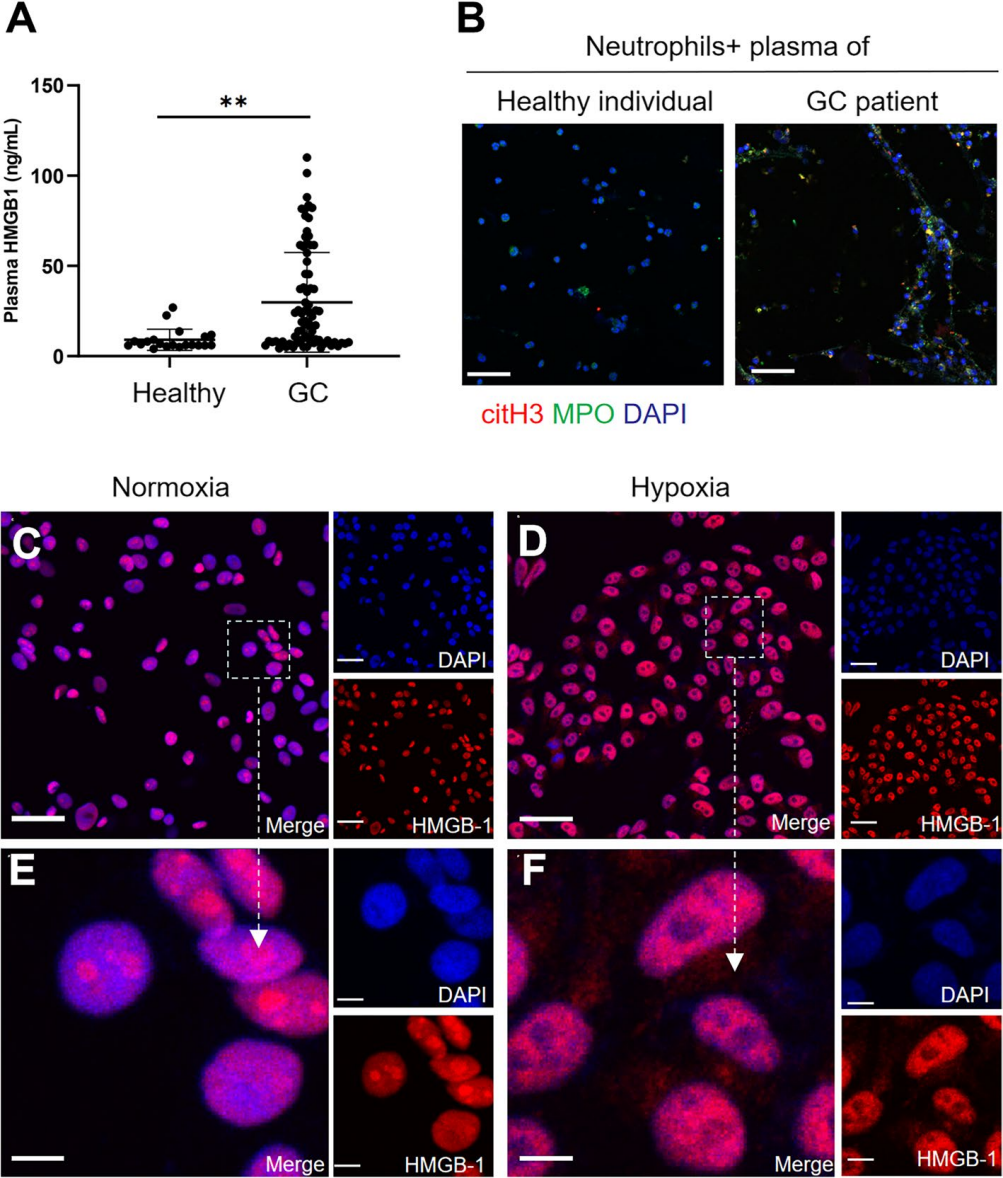
HMGB1 was translocated to the cytoplasm from the nucleus when GC cells were cultured under hypoxic conditions. **A**, The levels of HMGB1 in the plasma of healthy individuals (*n* = 20) and GC patients (*n* = 80) were measured by ELISA. **B**, Neutrophils isolated from healthy individuals were cocultured with the plasma of healthy individuals or GC patients, and NET formation was detected with MPO and citH3 staining. Magnification: 20 × . Scale bars: 50 μm. **C** and** D**, The localization of HMGB1 in GC cells under normoxia and hypoxia was determined by IF staining. Magnification: 20 × . Scale bars: 50 μm.** E** and** F**, Magnified (63x) image of HMGB1 localization in CC cells under normoxic and hypoxic conditions. Scale bars: 10 μm. All values are the means ± SDs. **P < 0.01

In addition, we measured the plasma HMGB1 levels in GC patients and healthy individuals and found that GC patients showed significantly increased levels of plasma HMGB1 compared with healthy individuals (Fig. 5A). Furthermore, we cocultured neutrophils with plasma from GC patients and healthy individuals and found that plasma from patients with GC but not plasma from healthy individuals triggered robust NET formation (Fig. 5B). To determine whether hypoxia plays a role in inducing the expression or translocation of HMGB1 in GC cells, AGS cells were cultured under normoxic (20% O_2_) or hypoxic (1% O_2_) conditions. Under normoxia, HMGB1 remained localized predominantly in the nucleus. After exposure to hypoxia, the expression of HMGB1 was increased in the cytoplasm (Fig. 5C-F). These findings indicate that hypoxia leads to the nuclear-to-cytoplasmic translocation of HMGB1 in GC cells and mediates NET formation.

### HMGB1 mediates the formation of NETs via the TLR4/p38 MAPK pathway in neutrophils

**Fig. 6:**
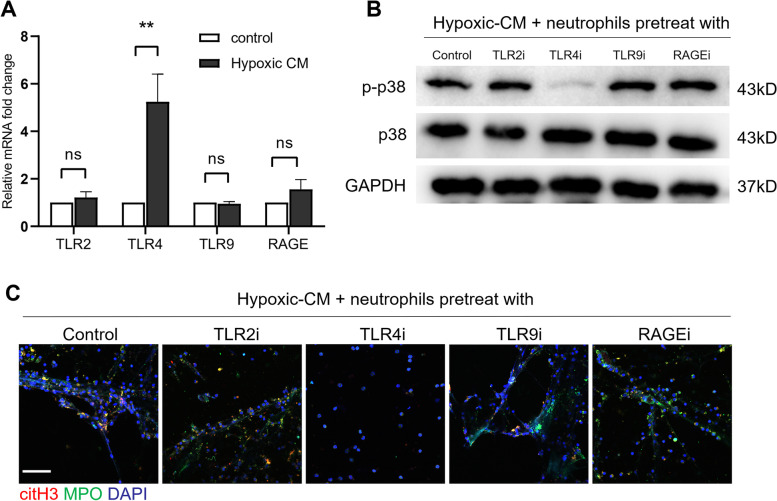
The hypoxic microenvironment induced NET formation through the TLR4/p38 MAPK pathway in neutrophils. **A**, The mRNA expression of TLR2, TLR4, TLR9 and RAGE in neutrophils treated with hypoxic-CM from GC cells was measured by qRT‒PCR. **B**, Neutrophils isolated from healthy individuals were pretreated with inhibitors of TLR2, TLR4, TLR9 and RAGE for 1 h prior to coculture with hypoxic-CM from GC cells, and the p-p38 level in neutrophils was measured by western blotting. **C**, NET formation was detected with MPO and citH3 staining. Magnification: 20 × . Scale bars: 50 μm. All values are the means ± SDs. ns = not significant, **P < 0.01

Since NET formation is markedly enhanced by extracellular HMGB1 under hypoxic conditions, we then explored the downstream signalling pathways of HMGB1 in NET formation by neutrophils. Toll-like receptors (TLRs) and the receptor for advanced glycation end products (RAGE) are important receptors of HMGB1 under inflammatory conditions [29], but it is unknown which factor take effect on the formation of NETs in the hypoxic microenvironment of GC. Thus, we determined the levels of TLR2, TLR4, TLR9 and RAGE mRNA in neutrophils treated with hypoxic-CM from AGS cells by qRT‒PCR. Notably, the mRNA level of only TLR4 was significantly increased in hypoxic-CM-treated neutrophils (Fig. 6A). Furthermore, we treated neutrophils with TLR2, TLR4, TLR9 or RAGE inhibitors prior to coculture with the hypoxic-CM of AGS cells. We found that the TLR4 inhibitor but not the inhibitors of TLR2, TLR9 and RAGE abrogated hypoxic-CM-induced p38 MAPK pathway activation in neutrophils and NET formation (Fig. 6B and C). Taken together, these results showed that hypoxia induced neutrophil activation and NET formation in the GC TIME through the HMGB1/TLR4/p38 MAPK signalling pathway.

### NETs in the tumour microenvironment are correlated with more rapid tumour growth in vivo

**Fig. 7:**
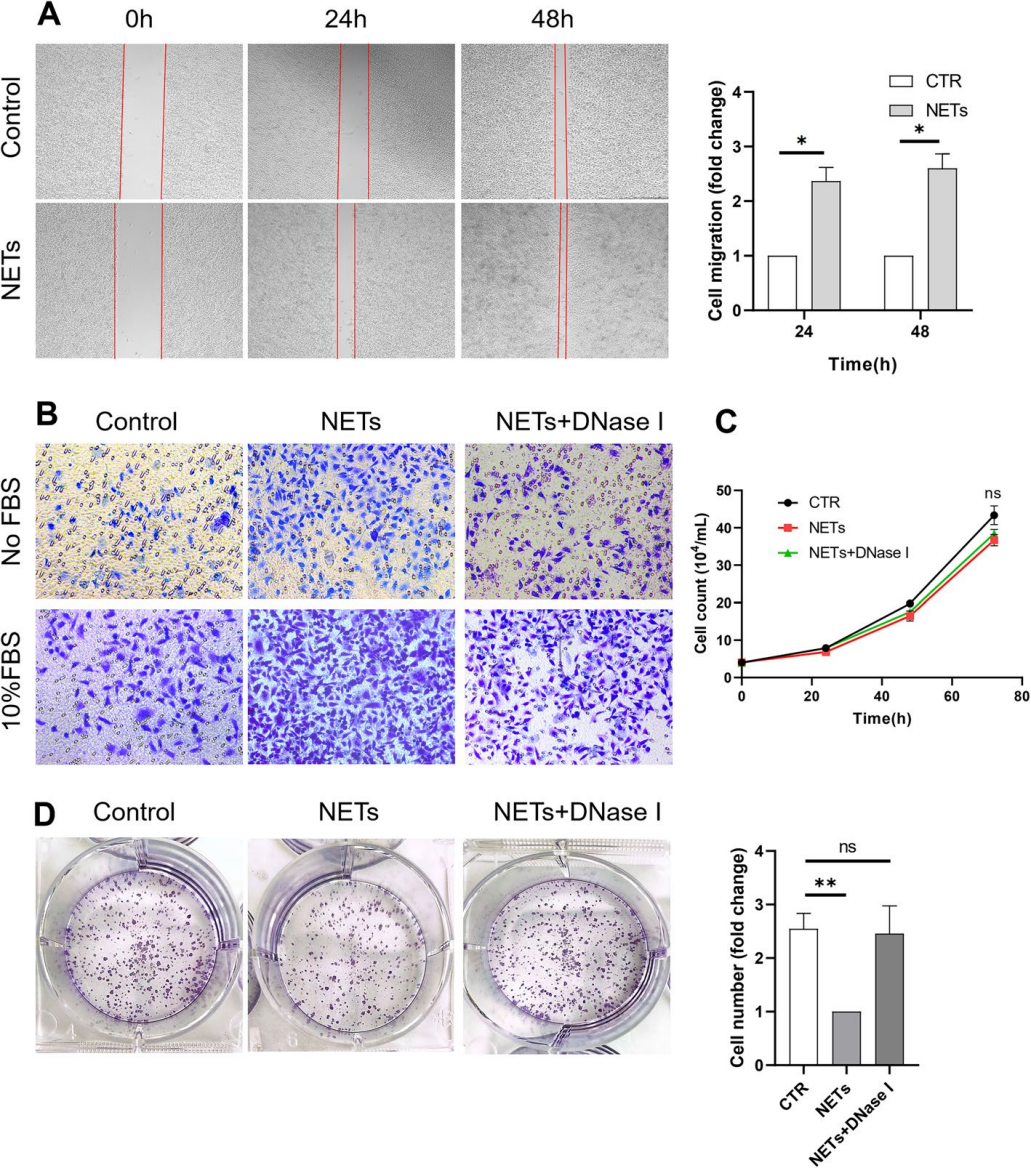
NETs promote GC cell invasion and migration but not proliferation. GC cell was pretreated with cell-free NETs and then the protumour effect of NETs was performed. **A** and** B**, The migration and invasion of GC cell pretreated with NETs was significantly increased. **C**, No difference of proliferation between control and NETs treated GC cell. **D**, NETs demonstrated cytotoxity on GC cell in colony-formation assay. All values are the means ± SDs. *P < 0.05, **P < 0.01

**Fig. 8:**
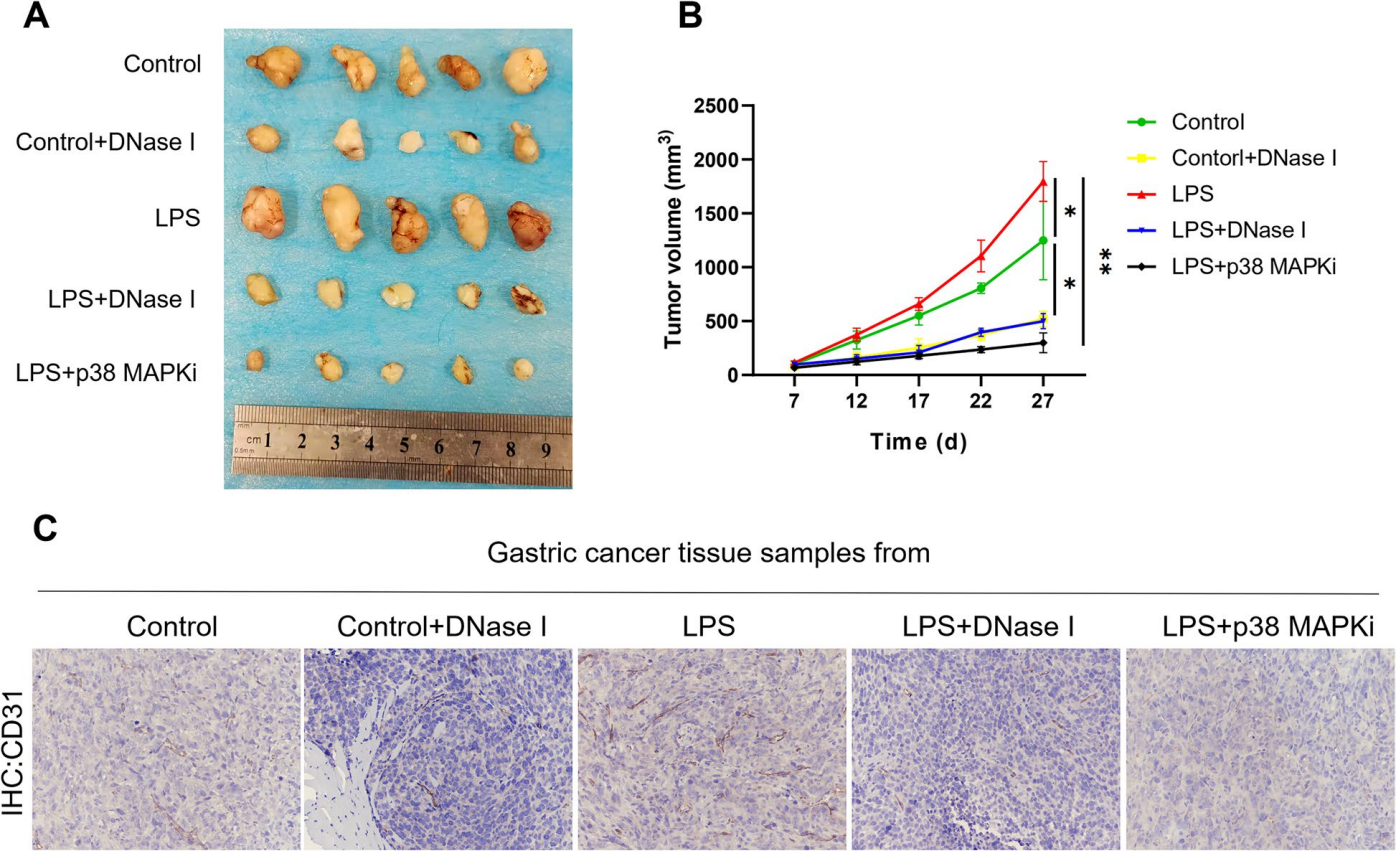
NETs accelerate tumour growth in vivo via angiogenesis. **A** and** B**, Subcutaneous AGS tumour growth was increased in the presence of NET-producing neutrophils from LPS-treated mice in vivo (*n* = 5 each group). **C**, Increased angiogenesis in tumours of LPS-treated mice compared to control mice. Representative images of CD31 staining are shown. Magnification: 20 × . Scale bars: 50 μm. All values are the means ± SDs. *P < 0.05, **P < 0.01

Having shown high levels of NETs in clinical samples from GC patients [18], we sought to investigate whether NETs have an effect on GC progression. We treated AGS cells with cell-free NETs and then assessed the protumour effect of NETs. As expected, treatment with NETs promoted the migration and invasion of AGS cells in vitro (Fig. 7A and B). Interestingly, the proliferation of AGS cells pretreated with cell-free NETs was not increased compared with that of control AGS cells (Fig. 7C), and NETs even demonstrated cytotoxicity in AGS cells in the colony formation assay (Fig. 7D), a pattern similar to their effects on pathogens, playing a “trap and kill” role to suppress cancer cell growth rather than promote tumour growth. However, the results of the in vitro cell proliferation assay were inconsistent with the clinical data. To determine the specific role of NETs in tumour growth in vivo, we next determined the rate of subcutaneous tumour growth in BALB/c nude mice injected with AGS cells. Of note, the rate of tumour growth was significantly increased in LPS-treated mice compared with control mice (Fig. 8A and B). In addition, the rate of tumour growth was markedly decreased when LPS-treated tumour-bearing mice were administered DNase I or a p38 MAPK signalling pathway inhibitor, which can inhibit the formation of NETs ex vivo, indicating that NETs can augment tumour growth in vivo.

The above studies showed conflicting results: there was no significant change in the proliferation of GC cells cocultured with NETs compared with that of control cells in the in vitro study, whereas the in vivo study demonstrated that NETs promote tumour growth, as the tumour volume was much larger in LPS-induced NET model mice subcutaneously implanted with GC cells than in the corresponding control mice. To further investigate the unclear effects of NETs on tumour growth, we examined CD31 expression in tumour samples from mice to explore whether angiogenesis, another of the most common causes of tumour growth augmentation, is involved in this process. IHC staining demonstrated that CD31 expression was much stronger in the tumour samples from LPS-treated mice than in those from control mice and that DNase I and the p38 MAPK signalling pathway inhibitor abolished this response (Fig. 8C), indicating that NETs accelerate GC growth by promoting angiogenesis rather than by directly increasing the proliferative capacity of GC cells.

## Discussion

In this study, we showed that the preoperative serum NET levels in GC patients were markedly increased compared with those in healthy individuals and were positively correlated with poor prognosis in patients with GC. In addition, histopathologic analysis of tissue samples from GC patients demonstrated the complex relationships between the hypoxic microenvironment and neutrophil infiltration as well as NET formation. Notably, the translocation of HMGB1 from the nucleus to the cytoplasm of GC cells under hypoxic conditions stimulated neutrophil activation and NET formation via the TLR4/p38 MAPK signalling pathway in neutrophils. Furthermore, NETs accelerated tumour growth by promoting angiogenesis rather than directly increasing the proliferative capacity of GC cells.

Inflammation is one of the recognized hallmarks of cancer [30]. Many studies have reported that cancer-related inflammation located at the site of the tumour is related mainly to the local immune response, which frequently precedes and contributes to tumour development [31, 32]. Cancer-related inflammation is a sustained and possibly inappropriate systemic response to malignant tumours and can lead to fever, sweating, weight loss and various other paraneoplastic symptoms. For example, tissue injury and necrosis, effects of external pathogens or autoimmune diseases can lead to local immune responses, thereby mediating the initiation and promotion of carcinogenesis [7]. Obviously, the relationship between the host immune and inflammatory responses in tumours is complex, and the key process involved in this response is far from being fully understood. Cytokines, chemokines and other inflammatory proteins secreted from tumour or immune cells coordinate the intercellular communication in the TIME [33, 34]. In addition, these inflammatory reactions provide a reasonable intervention target for tumour treatment with some nonselective, inexpensive and low-toxicity antibiotics, which have the potential to improve tumour prognosis.

Hypoxia is a common feature of solid tumours and is related to poor prognosis [33, 35, 36]. The ability of tumour cells to adapt to changes in oxygen tension in a hypoxic microenvironment is crucial to the progression of tumours. A hypoxic microenvironment may promote the acquisition of more aggressive phenotypes by cancer cells, such as migratory, invasive and metastatic behaviours as well as chemoresistance, by changing the heterogeneity and plasticity of tumours, which can accelerate cellular adaptation under hypoxic conditions. Much evidence has shown that highly proliferative tumours quickly outgrow their blood supply, resulting in a hypoxic and necrotic tumour core [37]. Subsequent cell death releases DAMPs, which cause immune cell infiltration into hypoxic and necrotic areas; these cells, such as tumour-associated macrophages (TAMs), tumour-associated fibroblasts (TABs) and tumour-associated neutrophils (TANs), then become components of the TIME [37–39]. In this study, we found that GC cells secreted chemokines such as CXCL1/KC, CXCL2/MIP2 and HMGB1 under hypoxic conditions to mediate neutrophil infiltration, which may explain the role of TANs in the TIME.

Neutrophils account for a large portion of the inflammatory cells in the TIME of various tumours [40]. In fact, both the antitumour and protumour characteristics of neutrophils have been confirmed. Despite their normal function of killing pathogens, accumulating evidence has shown the crucial role of TANs in tumour proliferation, invasion and metastasis. Since the mechanism of NETs was first described in the inflammatory response, novel insight into the protumour role of neutrophils via NETosis has attracted the attention of researchers [41]. Many studies have shown that NETs are involved in cancer progression by promoting cancer cell proliferation, invasion, and metastasis and even angiogenesis and cancer-associated thrombosis (CAT) in various tumours [15, 26, 42, 43]. In this study, histopathologic analysis found that infiltrating neutrophils and NETs accumulated in the hypoxic microenvironment of GC, indicating the complex relationship between hypoxia and NETs in the TIME.

Recent studies have shown that NETs are located in liver tissue under I/R conditions and promote CRC cell hepatic metastasis in animal models [26]. Of note, hypoxic-CM from CRC cells stimulated normal neutrophils to release NETs. Previous study showed that neutrophils isolated from GC patients were more disposed to NET release than were control neutrophils when incubated with a low concentration of PMA, indicating that neutrophils in the TIME of GC are already activated [15]. In this study, we found that hypoxic-CM from GC cells had the ability to induce NET release from control neutrophils; even though normoxic-CM demonstrated an ability to induce NET formation, this ability was markedly decreased compared with that of hypoxic-CM. To determine the molecular mechanism of NET formation in the hypoxic microenvironment of GC, we cocultured neutrophils with hypoxic-CM, and the activation of signalling pathways was evaluated by western blotting. We proved that hypoxic-CM from GC cells mediated NET formation via the p38 MAPK signalling pathway. Furthermore, we found that HMGB1 was translocated to the cytoplasm from the nucleus of GC cells under hypoxic conditions and had the ability to stimulate NET release from neutrophils, which could be reversed by treatment with HMGB1 siRNA and an HMGB1 antagonist. In addition, we analysed the downstream pathway of HMGB1 and found that the mRNA level of TLR4 was significantly increased compared with those of TLR2, TLR9 and RAGE in neutrophils treated with hypoxic-CM from GC cells. Inhibition of TLR4 in hypoxic-CM using a specific antagonist blocked the activation of the p38 MAPK pathway in neutrophils, indicating that NET formation in the GC TIME is mediated through the HMGB1/TLR4/p38 MAPK pathway.

Based on the above experiments, we clearly elucidated the mechanism of NET formation in the GC TIME; thus, we continued to explore the role of NETs in the progression of GC. Accumulating evidence has suggested that NETs can trap cancer cells in an early stage and promote metastasis [9, 44]. Consistent with these studies, we also found that NETs can directly induce GC cell invasion and migration. However, the role of NETs in promoting the proliferation of cancer cells is uncertain. Some studies have shown that NETs can directly stimulate cancer cell proliferation, while others did not show this effect, perhaps related to the different types of tumours [16, 20]. Robust cancer cell proliferation is essential to the uncontrolled growth phenotype of malignant tumours. In this study, we found that NETs did not directly increase the proliferation of GC cells and even had a cytotoxic effect on GC cells, playing a “trap and kill” role, suggesting that NETs may inhibit tumour growth by killing cancer cells via a cytotoxicity pattern similar to that used to eliminate pathogens. Therefore, we established an LPS-induced NET model and a subcutaneous xenograft tumour model in athymic BALB/c nude mice to investigate this conflicting effect of NETs on GC cells. Interestingly, the results showed that tumour-bearing mice in the LPS-induced NET model demonstrated a higher tumour growth rate than the corresponding control tumour-bearing mice, indicating that NETs can augment tumour growth in vivo. Of note, the IHC images of tumour tissues from tumour-bearing mice revealed that the expression of CD31 was markedly increased in LPS-induced NET model mice compared with control mice; CD31 expression is representative of angiogenesis, indicating another exacerbated phenotype of tumour progression. In addition, we treated animals with DNase I and a p38 MAPK pathway inhibitor daily via intraperitoneal injection and found that tumour growth was significantly decreased. Of note, LPS-induced NET model mice treated with the p38 MAPK pathway inhibitor exhibited lower tumour volumes than those treated with DNase I, suggesting that not only NET-DNA but also other components of NETs have the ability to accelerate tumour growth.

Taken together, our results clearly revealed the mechanism of NET formation in the GC TIME and further demonstrated the important role of NETs in the augmentation of GC growth. Targeting NETs may constitute a treatment strategy in patients with GC. In the future, we will continue to deeply study the effects of common anti-inflammatory drugs on neutrophil infiltration and NET formation in the GC TIME, highlighting a new use for old drugs in GC treatment.

## Conclusions

Our findings demonstrated that a high level of NETs was positively correlated with poor prognosis in patients with GC. The hypoxic microenvironment of GC induced neutrophil infiltration and NET formation. HMGB1 translocated to the cytoplasm from the nucleus of GC cells to mediate NET formation in the TIME via the TLR4/p38 MAPK pathway in neutrophils. Notably, the tumour volumes in LPS-induced NET model mice were significantly increased compared with those in control mice, and targeting NETs with DNase I and a p38 MAPK pathway inhibitor blocked the augmentation of tumour growth.

## Methods

### Patient data and tissue specimens

Patients diagnosed with primary GC who underwent radical D2 resection and healthy individuals seen at the First Affiliated Hospital of Jiamusi University between 2017 and 2019 were enrolled in this study. Patients who were underage (< 18 years), on antitumour treatment before surgical treatment or had underlying complications such as endocrine, cardiovascular, haematological or infectious disease or other cancers were excluded from the study. Blood samples, tumour and adjacent normal tissue samples were obtained from GC patients who consented to this study. All patients were disease-free after surgery and had a minimum of one year of follow-up. Finally, eighty GC patients and twenty healthy individuals were enrolled in this study. Overall survival (OS) was calculated from the date of pathological diagnosis to the date of death, and disease-free survival (DFS) was calculated from the date of radical resection to the date of disease recurrence or death. Human neutrophils and platelet-free plasma (PFP) were isolated by density gradient centrifugation as previously described [18]. The protocol for this study was approved by the ethics committee of the First Affiliated Hospital of Jiamusi University.

### Cell culture and transfection and preparation of hypoxic-conditioned medium (CM)

The human primary GC cell lines AGS and HGC-27 were purchased from Procell Life Science&Technology Co.,Ltd. (Wuhan, China). GC cells were cultured in RPMI 1640 medium (Gibco, USA) supplemented with 10% foetal bovine serum (FBS; Gibco, USA) and 1% penicillin‒streptomycin solution (Beyotime, Beijing, China). Incubation was performed at 37 °C in 5% CO_2_ with 1% O_2_ or 20% O_2_ in a humidified environment. For siRNA transfection, the cells were transfected with specific siRNAs using Lipofectamine™ 3000 (Invitrogen, USA) according to the manufacturer’s instructions. The oligonucleotide sequences of the siRNAs were listed below:

- HMGB1, F: 5'-GGCCCGUUAUGAAAGAGAATT-3', R: 5'-UUCUCUUUCAUAACGGGCCTT-3'; Control, F: 5'-UUCUCCGAACGUGUCACGUTT-3', R: 5'-ACGUGACACGUUCGGAGAATT-3'.

Hypoxic conditions medium (hypoxic-CM) of GC cells were prepared when cells cultured to 90% confluence at 37 °C under 20%O_2_ and 5% CO_2_ and recultured for 6 h, 12 h or 24 h in medium without FBS at 37 °C under 1% O_2_ and 5% CO_2_. The supernatant was collected and centrifuged at 500 × g for 10 min at 4 ℃ to remove cell debris for subsequent experiments.

### Treatment of neutrophils

Neutrophils isolated from healthy individuals were suspended in RPMI 1640 medium supplemented with 1% FBS and incubated with normoxic-CM, hypoxic-CM, hypoxic-CM (HMGB1 siRNA), recombinant human HMGB1, recombinant human chemokine (C-X-C motif) ligand 1 (CXCL1) or recombinant human CXCL2 for 4 h. For experiments that used inhibitors, neutrophils were pretreated with inhibitors for 1 h prior to the addition of hypoxic-CM from GC cells. The inhibitors used in this study included an HMGB1 antagonist (HY-N0184, MCE, USA), a p38 MAPK pathway inhibitor (HY-12839, MCE, USA), a p65 NF-KB pathway inhibitor (HY-138537, MCE, USA), an ERK pathway inhibitor (HY-112287, MCE, USA), a TLR2 antagonist (HY-112146, MCE, USA), a TLR4 antagonist (HY-107575, MCE, USA), a TLR9 antagonist (HY-131952, MCE, USA), and a RAGE antagonist (HY-P2268).

### ELISA

The amounts of MPO-DNA complexes in human and mouse plasma were quantified using capture ELISA as previously described [45]. The chemokine levels in the CM of normoxic or hypoxic GC cells at different times were measured using capture ELISA (CXCL1/KC, CXCL2/MIP2 and HMGB1 ELISA kits, Jingkbio, Shanghai, China) according to the manufacturer’s instructions.

### Animal models

The animal protocol was designed to minimize pain or discomfort to the animals. Male BALB/c nude mice (6–7 weeks old, weighing 18–22 g) were purchased from Beijing Vital River Laboratory Animal Technology, and housed in a specific pathogen-free mouse facility. All procedures were approved by the Animal Care and Use Committee of the First Affiliated Hospital of Jiamusi University. Then, we established a well-used lipopolysaccharide (LPS)-induced NET model as previously described [25]. Briefly, LPS (10 μg/mouse, Beijing) was intraperitoneally injected to induce systemic inflammation in BALB/c nude mice. Deoxyribonuclease I (DNase I, 100 U/mouse, Roche) or a p38 MAPK signalling pathway inhibitor (1 mg/kg, MCE, USA) was injected intraperitoneally 24 h before injection of LPS as the NET inhibitor.

In the xenograft models, BALB/c nude mice were injected with AGS cells (2 × 10^6^ cells/mouse) subcutaneously into the right axilla. DNase I (100 U/mouse, Roche) and the p38 MAPK signalling pathway inhibitor (1 mg/kg, MCE, USA) were administered intraperitoneally daily after AGS injection. Mice were sacrificed at day 27 after GC cells injection. Tumour volumes were calculated by measuring the length (L) and width (W) of the tumours: Tumour volume = π/6 × L × W^2^.

### Neutrophil chemotaxis assay

Neutrophils (1 × 10^5^ cells) isolated from healthy individuals were seeded in RPMI 1640 medium supplemented with 1% FBS in the upper chamber of the Transwell apparatus. The lower chamber contained normal CM or hypoxic-CM from GC cells collected at different times. After the cells migrated for 3 h, nonmigrated cells were removed by scraping with cotton-tipped applicators. The migrated cells on the underside of the chamber membranes were fixed, stained and counted under a microscope.

### Cell proliferation and colony formation assay

For the cell proliferation assay, GC cell monolayers were incubated with cell-free NETs in the presence or absence of DNase I for 12 h at 37 °C in 5% CO_2_ and 20% O_2_ in a humidified chamber. Then, treated GC cells (2 × 10^4^ cells/mL, 2 mL/well) were seeded in 12-well plates and cultured with 10% FBS medium and counted using a haemocytometer at the indicated endpoints.

For the colony formation assay, treated GC cells (1 × 10^3^) were seeded in 6-well plates at 37 ℃ in a 5% CO_2_ chamber. The medium was refreshed every 3 days for approximately 10 days, and the colonies were fixed, stained with 1% crystal violet and finally counted.

### Cell invasion and wound healing assays

The cell invasion assay was performed with the Boyden chamber invasion system as previously described [46]. Briefly, a Boyden chamber (Corning, USA) containing 8 μm pores was coated with Matrigel (Corning, USA). Human GC cells were cultured in the absence or presence of cell-free NETs (0.5 μg DNA/mL) for 12 h. Then, cells (4 × 10^4^) were seeded into the upper chamber, and medium without FBS or with 2% or 10% FBS was seeded into the lower chamber. After 20 h of incubation at 37 ℃ in an atmosphere of 5% CO_2_ and 95% air, nonmigrated cells were removed by scraping with cotton-tipped applicators. The migrated cells on the underside of the chamber membranes were fixed, stained and counted under a microscope.

For the wound healing assay, GC cells were stimulated with cell-free NETs for 12 h at 37 ℃ and then digested and seeded in 6-well plates with medium containing 10% FBS. When the GC cells were approximately 90% confluent, a 200 μl sterile pipette tip was used to scratch the cell surface, followed by three washes with PBS. Thereafter, the cells were incubated in medium supplemented with 1% FBS. Images of the scratch were acquired at different time points under a microscope. The cell migration rate was calculated as follows: (width at 0 h–width at different time points)/width at 0 h.

### Western blot assay

Western blotting was performed as previously described [47]. Briefly, proteins were extracted from tissue samples or neutrophils using RIPA lysis buffer containing proteinase inhibitors. Equal amounts of protein (20 μg) were separated by 10% or 12% SDS‒PAGE. Following electrophoresis, proteins were transferred to a PVDF membrane, blocked in 5% nonfat milk, and incubated with primary antibodies at 4 ℃ overnight. Antibodies against HIF-1α and citH3 were purchased from Abcam (UK), and antibodies against ERK1/2, p-ERK1/2, p65 NF-кB, p-p65 NF-кB, p38 MAPK, p-p38 MAPK, Akt, p-Akt, STAT3, p-STAT3 and GAPDH were purchased from Affinity Technology (USA). After washing with TBST three times, the membrane was incubated with HRP-conjugated goat anti-rabbit or anti-mouse secondary antibodies (Bioworld Technology) at RT for 1 h. The protein bands were visualized by enhanced chemiluminescence and analysed with ImageJ software (National Institutes of Health, Bethesda, MD, USA). GAPDH served as the loading control.

### Real-time quantitative PCR

Total RNA was extracted from GC cells and neutrophils using TRIzol reagent (Roche, Switzerland), and 1 μg of RNA was reverse transcribed to cDNA by using reverse transcriptase (Thermo Fisher Scientific, USA). Real-time quantitative PCR was performed by using a SYBR Green I real-time detection kit (Thermo Fisher Scientific, USA) on a Bio-Rad CFX96 Detection System. Relative mRNA expression was normalized to β-actin expression. The primers used for amplification of the target genes are listed in supplementary Table 1.

### Preparation of NETs

Cell-free NETs were isolated from neutrophils of GC patients as previously described with slight modifications [48]. Briefly, neutrophils (1 × 10^7^ cells/ml) were cultured for 4 h at 37 °C in 5% CO_2_ in medium supplemented with 200 nM PMA (HY-18739, MCE, USA). The supernatant was centrifuged at 500 × g for 10 min at 4 °C to remove cell debris. Thereafter, the supernatant (NET-rich medium) was centrifuged at 12,000 × g for 15 min at 4 ℃. The resultant pellets (a mixture of chromatin and protein) were suspended in ice-cold 1 × PBS, and the DNA concentration in the medium obtained was measured using spectrophotometry (Biospec-nano, Japan).

### Immunohistochemical assay

Briefly, 4 μm thick paraffin-embedded tissue sections were deparaffinized, rehydrated, treated with 0.3% hydrogen peroxide, and subjected to antigen retrieval with heat induction for approximately 10 min. The following primary were used for IHC staining: rabbit anti-CD66b (1:200, Ab197678, UK) and rabbit anti-CD31 (1:200, Ab76533, UK). The tissues were then incubated with a horseradish peroxidase-conjugated goat anti-rabbit IgG secondary antibody (ZSGB-bio, Beijing, China). The slides were finally stained with 3,3´-diaminobenzidine (DAB substrate kit, ZSGB-Bio, Beijing, China) and counterstained with haematoxylin. The specimens were analysed under a light microscope (Nikon, Tokyo, Japan) by pathologists.

### Immunofluorescence staining

For tissue samples, 6 μm Optimal Cutting Temperature (OCT)-embedded tissue sections were fixed with ice-cold acetone for 15 min. The following primary were used for IF staining: rabbit anti-citH3 (1:500, Ab5103, UK) and mouse anti-MPO (1:500, Ab90810, UK). Tissues were then incubated with Alexa Fluor 594-conjugated goat anti-rabbit (1:500, Ab150080, UK) and Alexa Fluor 488-conjugated goat anti-mouse (1:500, Ab150113, UK) secondary antibodies.

For NET formation, neutrophils (5 × 10^5^ cells) isolated from healthy individuals were seeded and incubated in glass-bottom poly-L-lysine-coated 24-well plates for 1 h at 37 ℃ in a 5% CO_2_ chamber and treated as described above. To detect and quantify NETs, the samples were incubated first with rabbit anti-citH3 and mouse anti-MPO primary antibodies and then with fluorescent secondary antibodies. All images were acquired using a confocal microscope (Zeiss, LSM 800, Germany) and analysed with ImageJ software (National Institutes of Health, Bethesda, MD, USA).

### Statistical analysis

Clinical data were available for all GC patients and healthy individuals. The survival of these patients was analysed using the Kaplan‒Meier method and compared using the log-rank test. Cox proportional hazards regression analysis was used to determine the effect of plasma MPO-DNA on OS and DFS. For in vitro and in vivo experiments, data are expressed as the means ± standard deviations (SDs). Data were analysed to assess distribution normality. For normally distributed data, statistical significance was analysed using Student’s t test and one-way analysis of variance (ANOVA). For nonnormally distributed data, statistical significance was analysed using the Mann‒Whitney test and the Kruskal‒Wallis test. All analyses were performed using GraphPad Prism v. 8.0 and SPSS 22.0 statistical software. *P* < 0.05 was considered statistically significant.

## Supplementary Information

**Additional file 1: Supplementary Table 1.** The sequences of primers for qPCR.

## Data Availability

The datasets used and/or analysed during the current study are available from the corresponding author on reasonable request.

## Abbreviations

citH3: Citrulline histone 3
CAT: Cancer-associated thrombosis
CRC: Colorectal cancer
CM: Conditioned medium
CXCL1: Chemokine (C-X-C motif) ligand 1
EMT: Epithelial-mesenchymal transition
DFS: Disease-free survival
DLBCL: Diffuse large B-cell lymphoma
DAMPs: Damage-associated molecular patterns
GC: Gastric cancer
HIF-1α: Hypoxia-induced factor-1α
HMGB1: High mobility group box 1
I/R: Ischaemia/reperfusion
LPS: Lipopolysaccharide
MPO: Myeloperoxidase
NETs: Neutrophil extracellular traps
NE: Neutrophil elastase
OS: Overall survival
PFP: Platelet-free plasma
RAGE: Receptor for advanced glycation end product
TIME: Tumour immune microenvironment
TANs: Tumour-associated neutrophils
TAMs: Tumour-associated macrophages
TABs: Tumour-associated fibroblasts
TLR: Toll-like receptor
