# Supplementary material for: Neutrophil extracellular traps induced by the hypoxic microenvironment in gastric cancer augment tumour growth
Source: Cell Commun Signal. 2023 May 1;21:86. doi: 10.1186/s12964-023-01112-5 (PMC10152773; doi:10.1186/s12964-023-01112-5)
Supplement: Supplementary file 2 — Additional file 1: Supplementary Table 1. The sequences of primers for qPCR. [file 12964_2023_1112_MOESM1_ESM.docx]

**SupplementaryTable 1. The sequences of primers for qPCR**

| Gene | Sequence |
| --- | --- |
| β-actin | F: 5'-CACGAAACTACCTTCAACTCC-3'  R: 5'-CATACTCCTGCTTGCTGATC-3' |
| HMGB1 | F: 5'-GGCCCGUUAUGAAAGAGAATT-3'  R: 5'-UUCUCUUUCAUAACGGGCCTT-3' |
| CXCL1 | F: 5'- CCAAACCGAAGTCATAGCCA-3’  R: 5'- TGGGGACACCTTTTAGCATCT-3’ |
| CXCL2 | F: 5'-GCCCAGACAGAAGTCATAGCC-3'  R: 5'-TCTTTGGTTCTTCCGTTGAGG-3' |
| TLR2 | F: 5'-ATCCTCCAATCAGGCTTCTCT-3'  R: 5'-GGACAGGTCAAGGCTTTTTACA-3' |
| TLR4 | F: 5'-AGACCTGTCCCTGAACCCTAT-3'  R: 5'-CGATGGACTTCTAAACCAGCCA-3' |
| TLR9 | F: 5'-CTGCCTTCCTACCCTGTGAG-3'  R: 5'-GGATGCGGTTGGAGGACAA-3' |
| RAGE | F: 5'-ATTTGGATCCCCGTCACTCTG-3'  R: 5'-GCCTGGCACCGGAAAATC-3' |
